# Study on the status and problems of teaching system of “medical advanced mathematics”: data based on a research of 11 universities in China

**DOI:** 10.1186/s12909-023-05012-7

**Published:** 2024-01-08

**Authors:** Jiangjie Sun, Tong Liu, Hui Li

**Affiliations:** 1 School of Management, Hefei University of Technology, Hefei, 230039 China; 2 School of Health Care Management, Anhui Medical University, Hefei, 230032 China

**Keywords:** Medical advanced mathematics, Curriculum system, Smart classroom, STC teaching mode

## Abstract

**Background and aim:**

Driven by Innovation 2.0 (the information age, the innovation form of the knowledge society), the form evolved by the Internet development, giving rise to economic and social development (“Internet +”). With this background, a novel approach is presented for fostering excellence in physicians, aligning with the contemporary demands of our era.

**Methods:**

Self-administered questionnaire was used to facilitate the collection of data on medical advanced mathematics course offerings, distribution of teaching hours of each major and the perception of the course teaching system in 11 medical universities in China. The distribution of course offerings in each major was analyzed, and one-sample t-test was conducted on the perspectives of course offerings, content settings (theoretical & practical), educational objectives, teaching reforms, and Synthetical Sensation (SS) of the curriculum system and educational model.

**Results:**

The study included various specialties such as clinical medicine, pharmacy, public health, health management, and life sciences, all of which offered advanced mathematics course. The content of medical mathematics textbooks was designed to meet the practical needs of relevant professions, and encompass online laboratory classes and social practice. However, a noticeable misalignment was observed between the content of medical mathematics courses and the realistic requirements of professions (t = -3.614~-3.018, *P* < 0.05). The perceived difference in the completeness of curriculum systems was not significantly apparent. There was a difference in the perception of the effectiveness of teaching reforms (*t = -4.485, P < 0.05*), and there was a difference in the perception of the synthesis of the educational model in all cases (*t = -5.067, P < 0.05*).

**Conclusion:**

There are localized differences in curricula, and the number of course hours is basically reasonable; course content needs to be updated; the implementation of course objectives is not in place; the curriculum system can meet the needs of talent training; the innovation of the education model needs to be put into practice; and there are obvious differences in the comprehensive cognition of the teaching system and the education model. Based on the analysis of the problems, we build a new STC teaching mode with smart classroom based on “professional needs, practical needs and requirements for cultivating excellent physician talents”.

**Supplementary Information:**

The online version contains supplementary material available at 10.1186/s12909-023-05012-7.

## Introduction

The reform of medical advanced mathematics teaching primarily focuses on three aspects: investigating differences in subject attributes during learning and cognition, exploring education and teaching modes, and researching restructuring of curriculum systems. In 2019, the Ministry of Education of the People’s Republic of China issued the “Guidance on Strengthening the Construction and Application of Online Learning Spaces”, proposing to actively promote the development of “Internet + Education” [1]. Education in high school is the “end” of talent cultivation and talent export, and it is also a key part of cultivating students’ innovation spirit, entrepreneurial consciousness and creative ability. As early as 2014, Premier Keqiang Li issued the slogan of “Mass Entrepreneurship, Mass Innovation”, calling for the attention of the society and universities. However, the cultivation of innovative talents depends on the curriculum system and educational teaching mode of talent cultivation. This is particularly evident that the logical reasoning of mathematics courses can cultivate students’ rational thinking, rigorous attitude of thinking about problems, and the deductive process can foster creative cognition [2]. Hence, the reform of mathematics curriculum system and educational teaching mode in universities is indispensable for talent cultivation.

In the face of the ravages of COVID-19, the cultivation of physician excellence is urgent. The core embodiment of physician excellence is its ability to innovate. However, medical advanced mathematics courses can serve not only as the underpinning for various medical-related professional courses — such as university physics, chemistry, and medical statistics, but also as core education for medical students to develop mathematical thinking. Moreover, it is also the key for medical workers to innovate medical diagnostic technology, such as applying mathematical modeling thinking to construct the operation model of disease diagnostic equipment. Meanwhile, medical advanced mathematics has made great contributions to the development of biomathematics, life science and quantitative genetics, and plays an irreplaceable value in the field of medical research. Therefore, the teaching of medical advanced mathematics has theoretical and practical significance in the training of outstanding physicians.

The reform of medical advanced mathematics teaching is mainly reflected in three aspects: studying differences in learning and cognition towards the discipline attributes, developing education and teaching mode, and improving curriculum system. Studies on discipline attributes concluded that spatial ability and verbal comprehension may play an important role in higher mathematics [3]. Analyzing gender differences based on the data from the Beijing Education Quality Assessment [4], it was realized that males are disadvantaged at the bottom of the distribution of grades and are in a disadvantageous position. Additionally, the impact of gender differences seems to vary based on the differences in schools and regions [5]. Motivation theory can be applied to the pipeline to math (STEM) careers to improve students’ interest in math learning [6] and the role of emotions in students’ learning processes, etc. [7]. In the realm of education and teaching modes, online education methods such as Micro-lecture, MOOC, and SPOC have gained prominence both domestically and internationally [8–13]. These approaches have yielded diverse positive teaching outcomes. For instance, integration of computer technology, artificial intelligence, and software in smart classrooms has effectively enhanced the teaching of complex, theoretical, and abstract subjects. This, to a certain extent, has contributed to the improvement in the quality of education and teaching [14, 15]. The PBL teaching mode flexibly integrates the biological model in medical mathematics classrooms, which improves the limitations of the traditional education mode [16–18]. In terms of curriculum system reform, little is involved, and there are only a small number of studies on the reconstruction of higher mathematics curriculum system [19–21]. Throughout the country and abroad, there is a lack of research for medical mathematics curriculum systems, especially construction of medical mathematics teaching mode based on professional.

## Subjects and methods

### Sample and data collection

Taking advantage of the opportunity of the National Conference on New Liberal Arts Teaching Reform in 2021 and the preparation of the textbook of Applied Mathematics in Medical Imaging, it is convenient to select 11 medical universities in China that offer the course of advanced mathematics in medical science. The universities selected cover the region of Tianjin, Shandong, Shanxi, Heilongjiang, Hebei, Guangzhou, Jiangxi and Anhui. The school level includes the entire undergraduate batch of the college entrance examination. The research subjects were teachers in the medical universities under study. Criteria for inclusion of subjects include: a) voluntary participation in the survey; b) confirmed to have very clear information about the math curriculum and talent development in their school; c) high title/position is preferred; d) only one subject from each school is selected. Exclusion Criteria include: a) Those who have unclear information about math course offerings and talent development in their school; b) More than 2 subjects from the same school with lower titles/positions. The sample data is representative.

### Tool

Self-designed questionnaire was used in this research to assess the current status of education and teaching of medical advanced mathematics in China and the problems that exist. This refinement was based on the existing mathematics public curriculum construction system and the professional enrollment catalogs of medical universities in China. In the context of China’s medical higher education personnel training system, “perceived information” related to medical mathematics was extracted, encompassing three factors: theoretical and practical content setting, educational objectives, and curriculum system and teaching modes. The questionnaire included five items: W1, the contents of current medical mathematics textbooks can meet the need of professional requirements; W2, the reform of medical mathematics teaching fully meets the cultivation of medical talents and can realize the integration of teaching and research; W3, the contemporary process of teaching medical mathematics covers a certain number of on-site laboratory or social practice classes; W4, the current medical mathematics teaching content is out of touch with the actual need of professionals; W5, the current curriculum system of medical colleges is inadequate and needs to be further integrated. According to the subjects’ personal views, options were selected to indicate the degree of agreement or disagreement, with 1 for total disagreement; 2 for basic disagreement; 3 for neither agreement nor disagreement; 4 for basic agreement; and 5 for total agreement. To conduct expert consultation on the questionnaire, we invited 7 professionals, including teaching management experts, subject teaching experts, and experts in the integration of medical teaching and research. After repeated discussions and two rounds of revisions, the “Questionnaire for Research on the Offering of Mathematics Series Courses in Undergraduate Medical Universities” was formed. The name of the subject’s school and demographic information were added to the questionnaire.

### Statistical method

All questionnaires were reviewed by two graduate students and the data were entered using EpiData 3.1. Word cloud diagrams were employed to visually represent the shortcomings in the curriculum system construction. Python software was utilized to analyze the distribution of course offerings within each major. Additionally, SPSS software was applied to conduct one-sample t-tests on various perspectives including course offerings, content settings (theoretical and practical), educational objectives, teaching reforms, and comprehensive perceptions of the curriculum system and educational model. The statistical analysis aimed to examine the differences in the perceptions of teaching experiences among the subjects.

Building upon this foundation, a two-dimensional area diagram was used to explore the distribution of teaching hours of advanced mathematics courses offered by each major, and finally, a box plot was used to visualize the comprehensive perception of teaching system and mode of medical advanced mathematics.

## Results

### The current status of the curriculum system of advanced mathematics in medical universities

In order to understand the current status of the curriculum system of “medical advanced mathematics”, we have conducted a research on the curriculum of “medical advanced mathematics” and the number of corresponding class hours of the whole batch of universities enrolling undergraduates in the domestic college entrance examination, and results gained are as follows:

### Differences in curriculum offerings

**Fig. 1:**
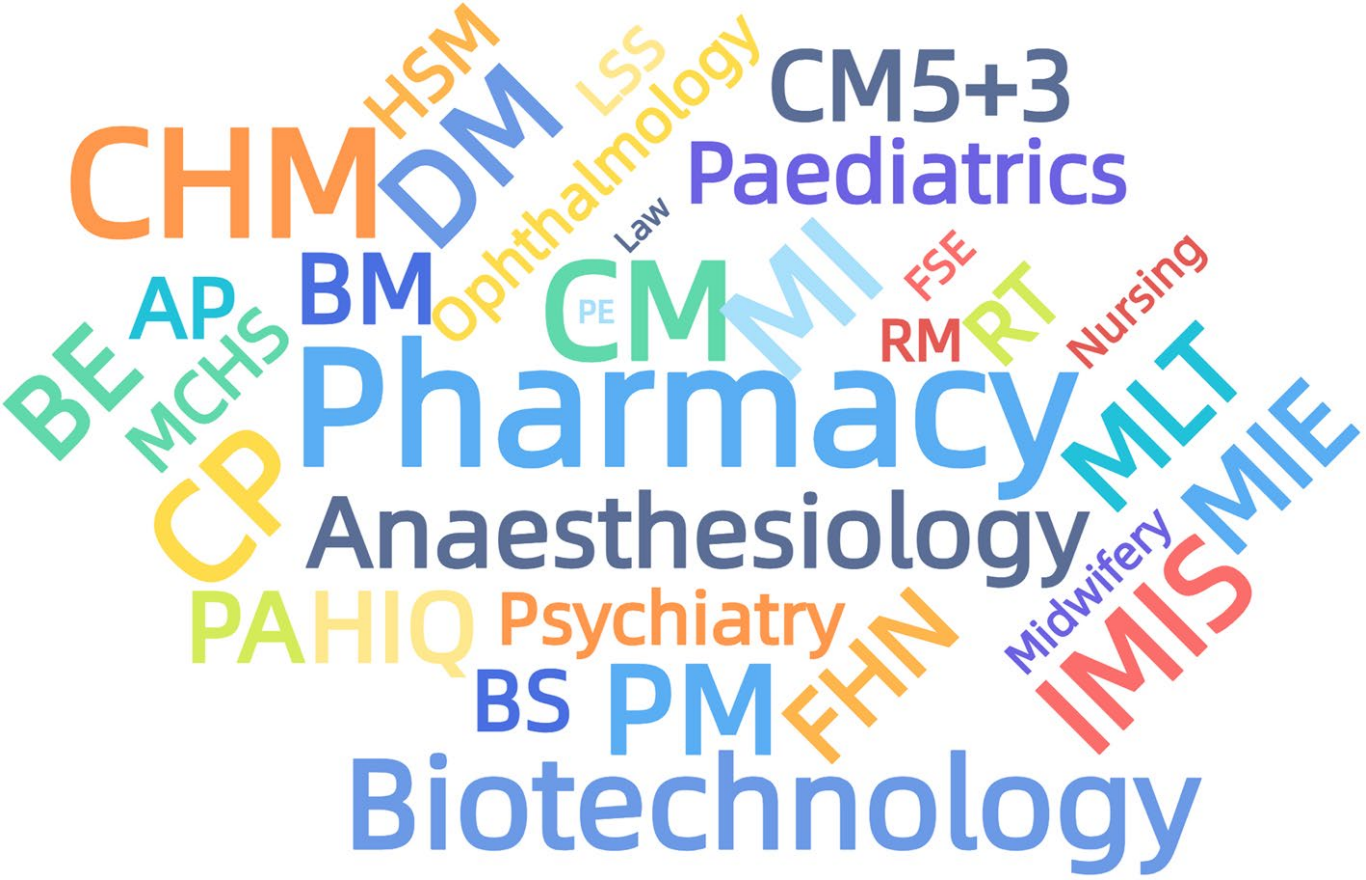
The word cloud map of the specialities offering advanced mathematics courses Notes: Clinical Medicine 5 + 3,CM5 + 3; Clinical Medicine, CM; Anaesthesiology; Medical Imaging, MI; Ophthalmology; Psychiatry; Radiological Medicine, RM; Paediatrics; Basic Medicine, BM; Dental Medicine, DM; Medical Laboratory Technology, MLT; Rehabilitation Therapy, RT; Health Inspection and Quarantine, HIQ; Food Hygiene and Nutrition, FHN; Food Science and Engineering, FSE; Preventive Medicine, PM; Maternal and Child Health Sciences, MCHS; Pharmacy; Pharmaceutical engineering, PE; Clinical Pharmacy, CP; Chinese Herbal Medicine, CHM; Nursing; Midwifery; Biological Sciences, BS; Biotechnology; Applied Psychology, AP; Public Administration, PA; Labour and social security, LSS; Health Services Management, HSM; Information Management and Information Systems, IMIS; Medical Information Engineering, MIE; Biomedical Engineering, BE

We extracted the information of “Advanced Mathematics” courses offered by each major in the selected colleges and universities and got the word cloud graph of majors who have offered “Advanced Mathematics” courses at present (see Fig. 1).

From Fig. 1, it can be seen that clinical medicine, pharmacy, public health, health management, life science and other majors offer advanced mathematics courses, presenting a general consistency of course offerings. Five universities offer advanced mathematics courses in AP. Southern Medical University and Qilu Medical College offer advanced mathematics in Nursing. Southern Medical University and Hebei Medical University offer advanced mathematics in Midwifery. Southern Medical University offers advanced mathematics courses in Forensic Medicine, which appears similar to Shanghai University of Finance and Economics offering 72 h of advanced mathematics courses in Psychology and Law.

### The curriculum is generally reasonable

**Fig. 2:**
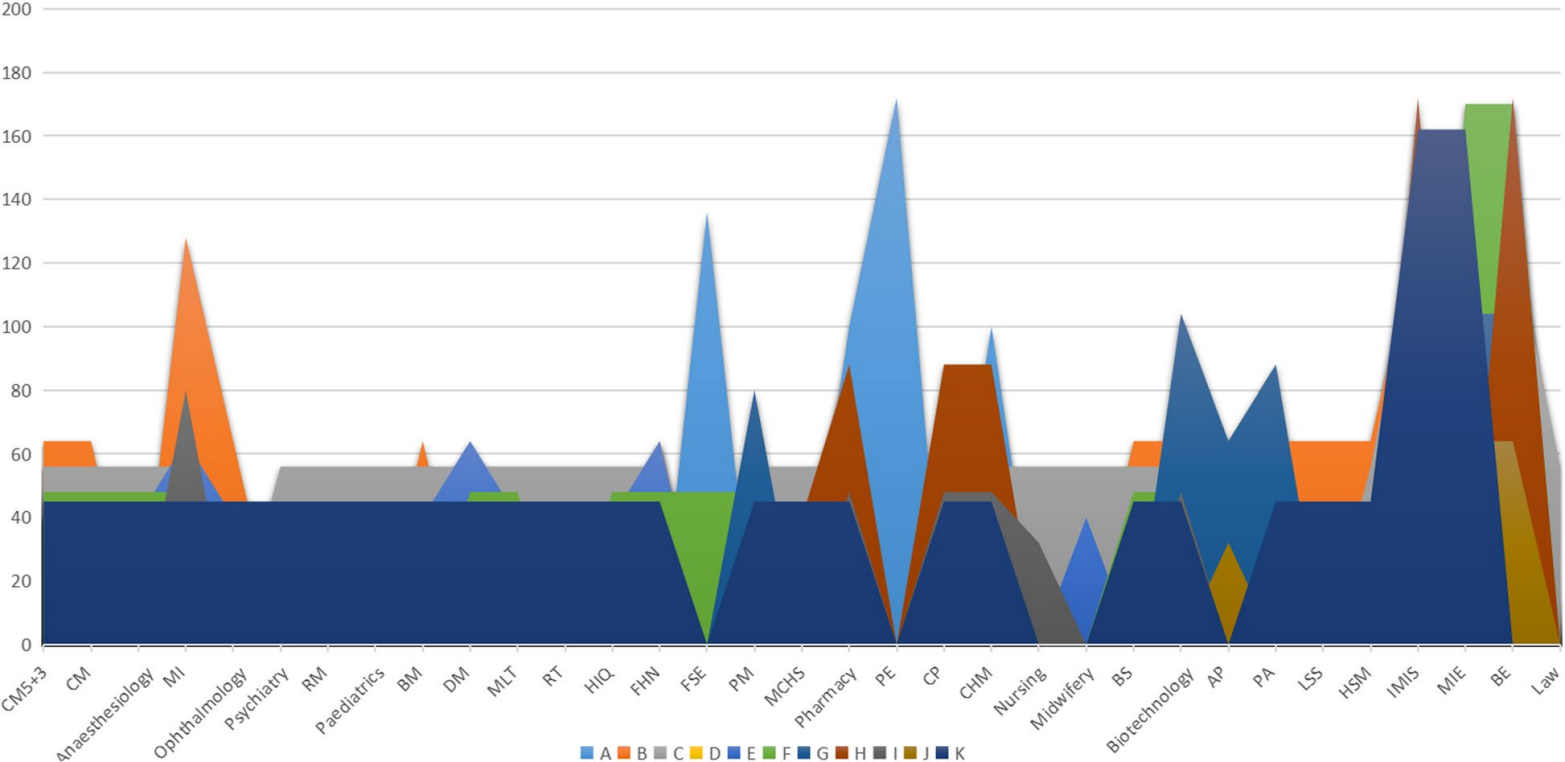
Two-dimensional area graph of the number of hours of advanced mathematics courses offered by each major Note: A, B, C… to show the number of hours of advanced mathematics courses offered by each major, K is the number of class hours in our university; The relevant notes are the same as Fig. 1

Based on the fact that domestic medical advanced mathematics textbooks chapter system and schedule are generally the same, it was realized that teaching content usually shows a positive relationship with teaching hours. Therefore, we used the advanced mathematics of each university to measure the richness of the knowledge system of the course. We used a two-dimensional area chart to give the span of advanced mathematics class time setting of each major in this study (see Fig. 2).

As can be seen from Fig. 2, the number of class hours offered by each major in our university is slightly lower, and only the number of class hours set for two majors (Information Management and Information Systems) are slightly higher. The overall number of class hours is around the median. It further shows that the course hours of advanced mathematics and teaching content system in our university are reasonable.

### Analysis of perceived data variability in the teaching system of advanced mathematics courses in medical universities

To enhance the understanding of the teaching of medical advanced mathematics in Chinese medical colleges and universities, we performed a one-sample t-test on various perspectives, including course offerings, content settings (theoretical and practical), educational objectives, teaching reforms, and comprehensive perceptions of curriculum system and educational model. The results of this analysis are presented in Table 1.

**Table 1:** Analysis of perceived data differences in medical advanced mathematics instructional systems

*Variables*	*N*	*Mean*	*SD*	*t*	*P*	*Test Value*
W1	11	3.00	1.095	-3.028	0.013	4
W2	11	2.82	0.874	-4.485	0.010	4
W3	11	2.45	1.440	-3.560	0.048	4
W4	11	3.18	0.751	-3.614	0.049	4
W5	11	3.82	1.168	-0.516	0.617	4
SS	11	3.06	0.559	-5.607	0.000	4

W1, W2, W3, W4, W5 have the connotations described earlier

*SS* Synthetical sensation

As can be seen from Table 1, there is a significant difference in the perception that the content of medical mathematics textbooks can meet the needs of professional practice (*t = -3.018, P ≤ 0.05*). As well as in the perception of the content of on-line experimental or social practice courses (*t = -3.560, P ≤ 0.05*). There is a significant disconnect between the content of medical mathematics teaching and the actual needs of professional talents (*t = -3.614, P < 0.05*), and there is no significant difference in the perception of whether the curriculum system is valid. There is a difference in the perception of the effectiveness of teaching reform (*t = -4.485, P < 0.05*), and in the perception of synthesis of educational models (*t = -5.067, P < 0.05*). It can be seen that the current implementation of the objectives of the medical advanced mathematics course is not in place, and the teaching system and educational model innovation is yet to be implemented.

### Visualization of the integrated perception of the teaching system and teaching model of advanced mathematics in medicine

**Fig. 3:**
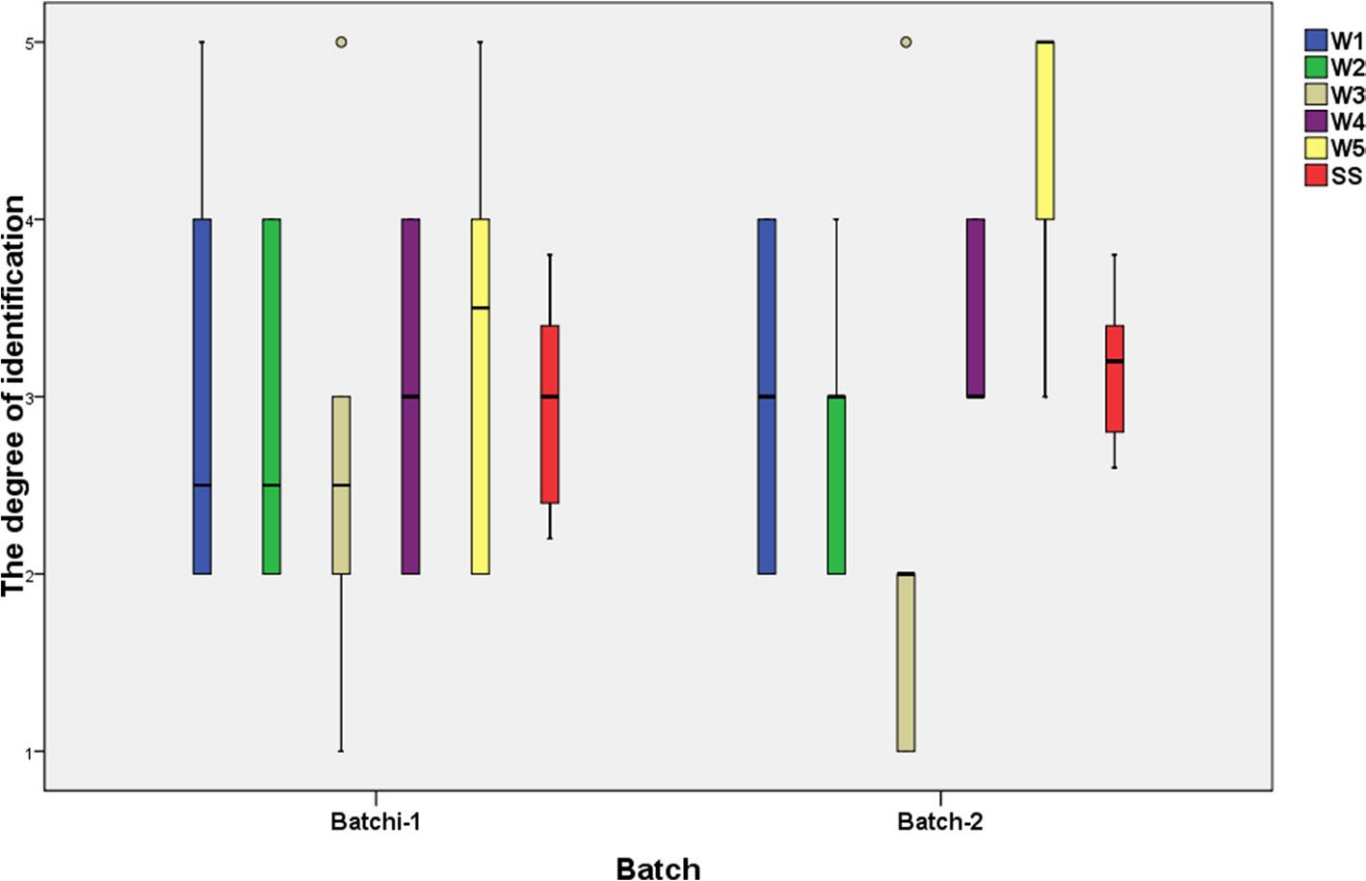
Teachers’ comprehensive perception of the medical advanced mathematics teaching system and educational models Note: The vertical axes 1, 2, 3, 4, and 5 indicate the degree of agreement with W1, W2, W3, W4, and W5 respectively, Larger values are more agreeable. Batch-i, medical colleges enrolled in batch i

In order to quickly understand the realities of education and teaching of higher mathematics in domestic medical universities, we visualized the data of the subjects’ comprehensive perception of the teaching system and education mode of higher mathematics in clinical medicine, and obtained Fig. 3.

As can be seen from Fig. 3, instructors believe that the content of current medical advanced mathematics textbooks can basically meet the needs of professional applications, and the cognitive differences among the full sample are not significant. The degree of integration of teaching and research of medical advanced mathematics is low, and the reform of teaching mode needs to be further promoted and implemented, especially in Batch-2 universities. In medical universities, there is a serious deficiency in the degree of experimental classes or practical classes offered by medical advanced mathematics in various majors, and almost none in Batch-2 universities. The disconnection between the teaching contents of Medical Mathematics and the needs of professional talents exists, but the performance is not obvious. There is a high degree of consensus among instructors that medical advanced mathematics curriculums can meet the needs of talent training. The level of teachers’ comprehensive perception of the system of teaching higher mathematics and the educational model is not high.

## Discussion

The results of this study show local differences in curriculum with universities offering advanced mathematics courses in Nursing, Midwifery and Forensic Medicine. One of the potential explanations for this observation could be that the leaders of these majors recognize and endorse the talent cultivation function of the mathematics discipline. For instance, they may acknowledge that the logical rigor inherent in the mathematics discipline plays a crucial role in developing the meticulous skills required for nurses’ practical service work, as highlighted by Sun et al. [22, 23]. Additionally, these leaders may see the value of mathematics in fostering logical reasoning and argumentative skills essential for disciplines such as forensic medicine. The existence of other reasons cannot be excluded.

The results of this study found that the curriculum objectives were not implemented. The Ministry of Education of the People’s Republic of China has issued an act on further strengthening the work of undergraduate teaching in universities. The document highlights the need to cultivate skills that enhance the coordinated development of students, with an emphasis on improving learning capabilities, practical skills, and innovation. However, the goal of the current higher mathematics courses in medical universities is still focusing on the knowledge ability, while neglecting the expansion and improvement of the practice ability. For example, we get from the interviews that most of the teaching process of medical mathematics only involves the explanation of concepts and definitions, the deduction of theorems and properties, or the introduction of algorithms, which all stay at the level of the transmission of knowledge. However, there is apparent ignorance in the needs of social practice, the need to cultivate talents, the transmission of mathematical thinking and the cultivation of the ability to apply and innovate. This is probably one of the main reasons as we can see from the current content system of the used math textbooks. Therefore, during the preparation of our textbook “Applied Mathematics for Medical Imaging” opened by People’s Health Publishing House in 2022, we have tried to overcome this shortcoming as much as possible, and added the application of knowledge thinking. Another possible reason could be that the opening school mathematics subject teachers are not equipped enough, belonging to the school marginal subject attributes of the hold; This may also stem from the individual ability differences in teachers. For teachers with strong teaching and research ability, a practical refinement of multidisciplinary problem modeling can be used through independent lesson planning [17], to further implementation of the course teaching objectives. Teachers with weak teaching and research ability highly rely on the existing teaching materials, basically just to achieve the function of the transmission of knowledge. Furthermore, there are some teachers facing retirement status, and they commonly lack the initiative of exploration. Certainly, other reasons should not be excluded.

The results of this study show that teaching hours of medical advanced mathematics courses are basically reasonable, and the curriculum system can meet the needs of talent training. The main reason comes from the fact that the textbook system of medical advanced mathematics teaching is mostly consistent, and the theoretical teaching content is basically similar. The secondary reason is that the teaching schedule design is not adjusted in time, and the revision of teaching materials is not strong, resulting in little change in the teaching hours of teaching programs. Combining these two reasons, it is understandable that there is no difference in class hours among current domestic universities of the same majors. The intrinsic theoretical nature of mathematics makes it challenging to articulate its direct application value in medical disciplines. Additionally, the extent to which it replaces the curriculum system is relatively limited. As a result, it is acceptable that “the perceived difference in whether the curriculum system is valid or not is not obvious”.

The results of this study found that the education model innovation needs to be encouraged. The domestic education model is generally similar to carry out education and teaching work, because it is mostly based on the textbook system. The phenomenon of aging knowledge of the textbook is more serious. In addition, medical mathematics is mostly in the framework of the public curriculum attributes of medical universities There is a serious shortage of the introduction of talents, and the structural nature of the teachers in the domestic medical universities have appeared in varying degrees of deficiencies. This could lead to the result that the concept of teacher education and teaching can not be aligned with the reality of the phenomenon of disconnect between the reform of teaching and practice in universities [11]. Simultaneously, the university’s educational evaluation system is not flawless, and there exists a notable lack of fairness in the public curriculum system. This imbalance contributes to a relative lack of enthusiasm among teachers, compounded by the less-than-optimal mathematical literacy of medical students. This situation results in a coupled frustration, as outlined in previous studies [11–13]. One primary contributing factor could be the demands of teaching innovations, such as Micro-lecture, MOOC, and SPOC, which necessitate investments in essential teaching equipment, educational resources, and ongoing human maintenance costs. However, in medical universities, Advanced Mathematics is a marginal subject in the public curriculum, and there is a lack of relevant resources to ensure its normal input, so that, in many cases, the so-called innovation is a “point-to-point” breakthrough, and it is difficult to form a system, and to promote a model like Engineering Mathematics or Science Mathematics. The secondary reason could be related to the human resources of the school. It needs to coordinate with multiple departments and members of the school, such as the first year of Advanced Mathematics, if the platform for high-quality courses is implemented, it is necessary to import the list of students into the system in a timely manner. However, operating authority involves multiple departments, which may bring a lot of administrative resistance to teachers who try to promote the model of innovation, and thus hinder the implementation of innovation. Other possible reasons can not be ruled out.

The results of this study showed that differences in the perceived synthesis of the teaching system and education model were significant, and the instructors believed that the content of current medical mathematics textbooks could basically meet the needs of professional practice. The differences in cognition of the full sample were not significant. The reason for this may be related to the consistency of the teaching system of medical advanced mathematics in each university [24–26]. The reason for the low degree of integration of textbooks and research in medical advanced mathematics teaching reform may be related to the fact that the medical advanced mathematics course is a public course, which is difficult to get high attention at the school level. this fundamental reason is especially obvious in the Batch-2 universities. In medical universities, there is a serious deficiency in the degree of offering laboratory or practical courses in medical advanced mathematics for all majors, and there is nearly no such course at all in the Batch-2 universities. One of the reasons may come from the shortage of senior faculty. Subsequently, there will be disconnection between the content of medical mathematics teaching and the needs of professional talents. The more obvious the problem is, the more faculty perceptions will resonate, that is, there is a high degree of agreement among the instructors that the advanced medical mathematics curriculum needs to be improved and integrated.

## Case-study

To solve the problems mentioned above and lay the foundation for cultivating excellent medical talents, we developed “teaching objectives, curriculum system, and STC teaching mode” from three perspectives, namely “professional needs, practical needs, and quality of talent cultivation” from three aspects of “professional needs, practical needs and talent training quality”.

### To orient teaching objectives to professional needs

It is necessary to break the original knowledge system of medical advanced mathematics, and develop medical professional course based on the professional application of Mathematics course. Demand task should be used for preparation of course content and teaching unit, to achieve “clarity of competency objectives, modularisation of curriculum structure, and actualisation of learning content”.

After conducting in-depth professional research and cooperating with professional course teachers in analyzing the courses of Health Statistics, Medical Imaging, Medical Physics and Chemistry, we analyzed the demand for mathematical knowledge in the pharmaceutical profession. Then we extracted typical demand tasks, integrated the tasks with relevant mathematical knowledge to form course modules, and then reconstructed the course system and transformed it into course contents according to students’ cognitive characteristics. Finally, a new system of “Introduction to medical advanced mathematics” course is formed.

The original curriculum competency goals were established according to the traditional Mathematics curriculum knowledge system, the core of which is to have strong arithmetic, thinking and logical reasoning skills. The systematic nature of knowledge is maintained, but the practical application of knowledge, especially in specialized courses, is neglected. According to the principle of “taking professional needs as the guide, taking practical needs as the purpose and taking quality of talents training as the key”, we establish the core competency of the new curriculum as “strong ability of mathematical operation and data analysis and the ability of using mathematical methods and tools to solve practical problems in medicine” .

### To reconstruct the curriculum system with the purpose of practical needs

All teachers in the research agree that the curriculum system of " medical advanced mathematics” needs to be improved and integrated, Based on the consensus, we have followed the principle of “the law of students’ growth and cognition and the development of students’ overall vocational ability” in order to achieve a “seamless connection” from mathematics to profession. We extract the “necessary and sufficient” contents from the original knowledge system and integrate them with the requirements of professional courses, and build a new curriculum system based on typical requirements. In order to facilitate the organization of teaching and learning, independent learning modules of the whole course are proposed according to the type of demand tasks.

The course content here is selected with emphasis on the demand for advanced mathematics in medicine and health professions, taking into account the sustainable development of students, to achieve the following three aspects: first, to meet the requirements of majors. By analyzing the requirements of the main professional courses of medicine and health on knowledge, methods and abilities of advanced mathematics, we closely link the teaching contents with the professional courses with the main line of requirements and tasks, so as to realize the “zero transition” from mathematics to professional courses. Second, professional knowledge leads to mathematical knowledge. Considering the actual learning conditions of students, the selection of content involves minimizing pure mathematical theory and emphasizing the introduction of mathematical knowledge through relevant professional examples. The training of creative thinking tools and methods is intensified, aiming to provide students with an experiential understanding of the significance of “methods being more important than knowledge.”

Third, to expand application ability. In order to improve students’ application ability, exercise students’ autonomy and creativity, the course content also includes: “application case study” training and “mathematical modeling” training in cooperation with the profession.

### To promote the STC teaching model with the quality of talent cultivation as the key

**Fig. 4:**
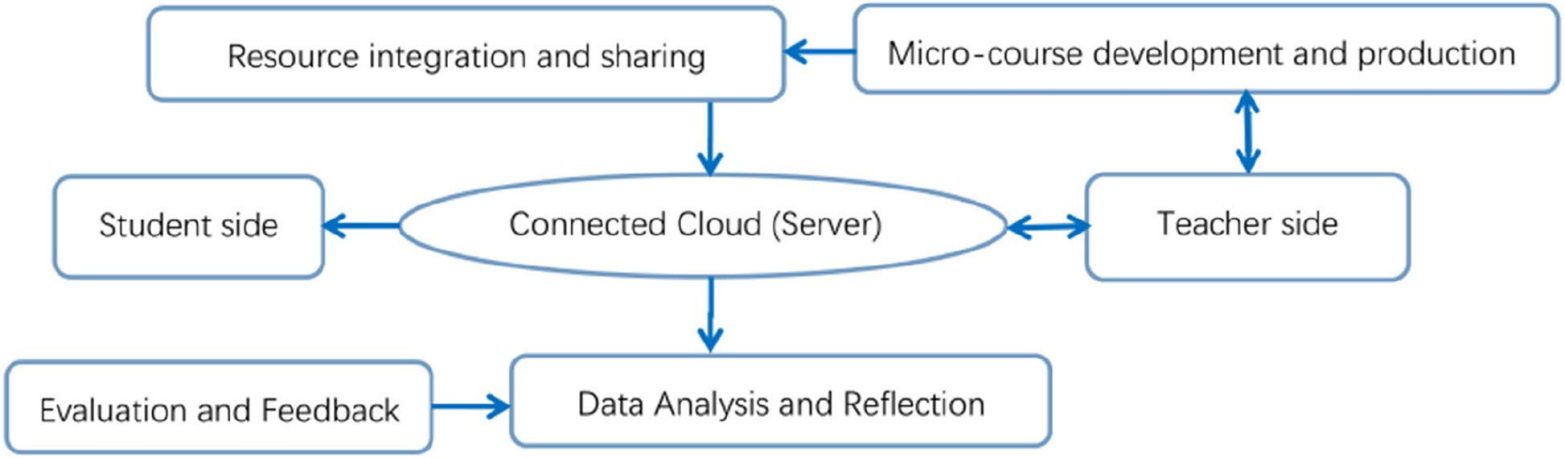
Framework of medical advanced mathematics smart classroom

In 2016, our group formally proposed the “STC teaching mode of Advanced Mathematics in medical science” based on “self-directed learning - traditional teaching - cross-collaborative learning”. S refers to the self-directed learning, which means the learning subject makes full use of the Internet quality resources to meet the personalized learning needs and standardized learning behavior under the condition of self-planning and deploying the learning progress; T refers to the traditional teaching, which means the learning subject receives the knowledge transmitted by the teacher in a fixed environment, and the main mode is offline teaching; C refers to cross-collaborative learning. In 2020, we completed the provincial-level smart classroom project of “medical advanced mathematics” and gained some experience in the construction path of “smart classroom” and STC teaching mode. In order to get rid of the constraints of traditional teaching, we have been promoting the STC teaching model of medical advanced mathematics, realizing the “smart classroom” of medical advanced mathematics with the integration of “student side, teacher side and interconnected cloud side” (see Fig. 4).

**Fig. 5:**
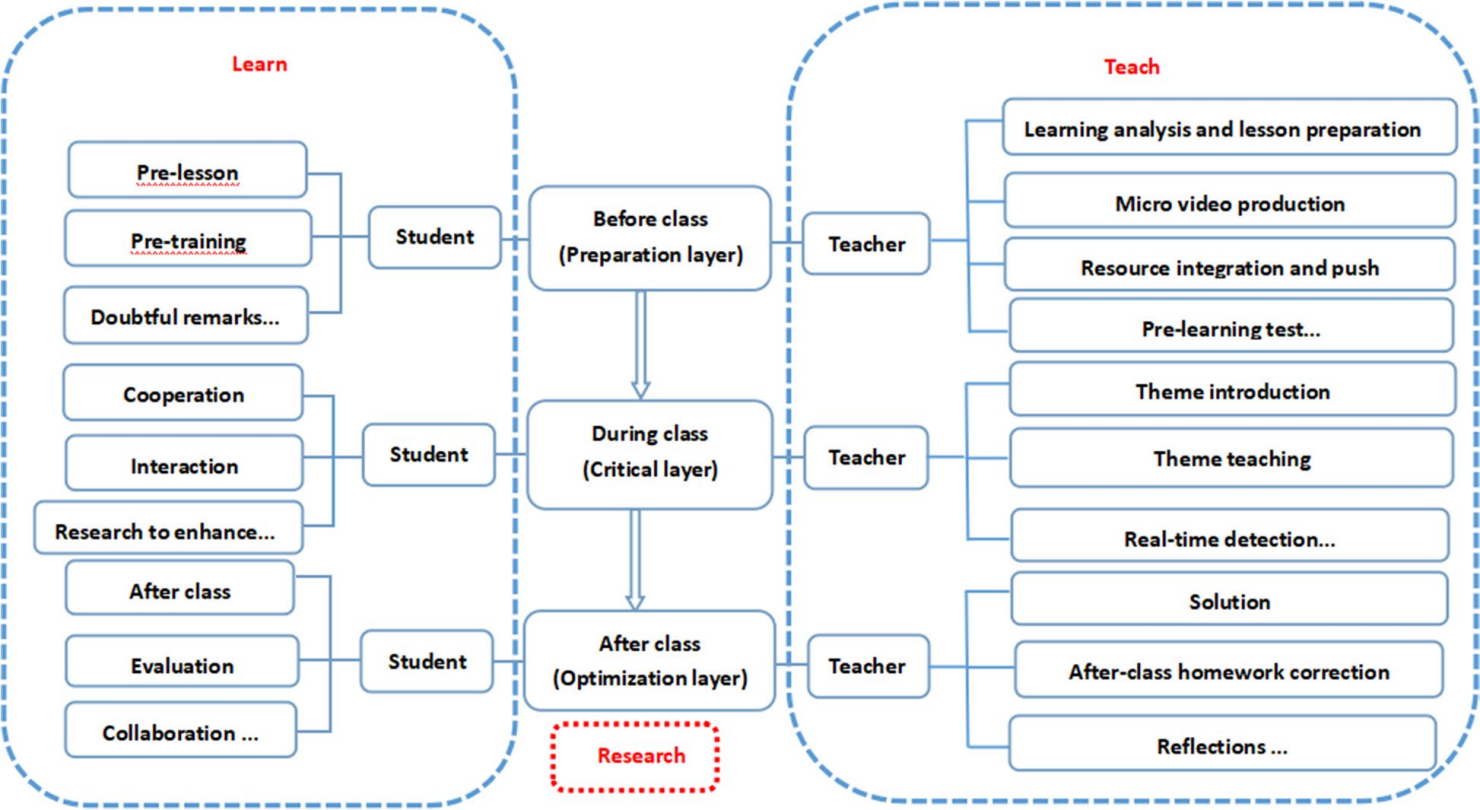
Flow chart of integration of teaching and research in STC of advanced mathematics for medical use

The practice of medical advanced mathematics smart classroom helps to integrate network resources, build a micro-course system of knowledge points, and reform in multiple dimensions such as from the modularization of teaching content, diversification of teaching methods, and temporalization of teaching venues. In essence, the implementation of smart classroom practices in medical advanced mathematics allows for flexibility beyond the constraints of original class schedules. This flexibility enables the dynamic alignment of teaching content with professional needs, thereby addressing and mitigating the issue of the disconnect between mathematics teaching content and the actual requirements of professional talents. Under the framework of smart classroom, “thematic micro-course construction” and “teaching and research integration” models for cultivating outstanding medical talents are carried out (see Fig. 5).

Based on the “professional needs, practical needs and requirements for cultivating excellent physician talents”, we have constructed a new model of STC teaching and research integration of medical advanced mathematics based on smart classroom. This model is expected to comprehensively solve the existing problems of cultivating excellent physicians in medical advanced mathematics. This can also be proved from our practical data. The STC teaching model of medical advanced mathematics was proposed in 2016, and in 2018, it won the third prize of teaching achievements of Anhui Provincial Department of Education. Furthermore, the micro-course construction won one first prize in provincial teaching competition, two special prizes, two special prizes in East China and two national second prizes in the process of domestic teaching competition. In 2020 we completed the construction of the reported smart classroom project, and won the second prize for teaching achievements of Anhui Provincial Department of Education in 2021. In terms of talent cultivation, we have achieved initial results, such as Jiang Yuanyuan, who is currently studying in Sun Zhongshan University, Yang Hangzhou from Shanghai Jiaotong University Hospital, Fang Jingyi from Fudan University Hospital, and Wang Ping from Shandong University, etc. Since the study of medical advanced mathematics course, undergraduate students participate in theme research workshop, in-depth research, and publication of ten SCI and EI papers and many papers in Chinese. Then a group of students, such as Wang Ping who is a research student in Shandong University and current students Zhang Qing, Ma Zuqing and Li Mengying, participated in publishing many SCI, EI and Chinese papers.

## Conclusion

China’s medical mathematics curriculum system basically meets the needs of talent cultivation; the innovation of the education mode of mathematics discipline is yet to be implemented; and the difference in comprehensive cognition of the teaching system and education mode is obvious. Actively promoting the new model of STC teaching based on smart classrooms is conducive to meeting professional demands and practice needs. It has practical significance for the cultivation of outstanding physician talents.

### Limitations

The data in the manuscript came from China only, and the lack of international data may lead to biased results. It is recommended that international data be added to subsequent studies to enhance the generalizability of the results to a certain extent.

### Supplementary Information

**Additional file 1.** Questionnaire for Research on offerings of mathematics series course.

## Data Availability

All data generated or analysed during this study are included in this published article.
