# Supplementary material for: Study on the status and problems of teaching system of “medical advanced mathematics”: data based on a research of 11 universities in China
Source: BMC Med Educ. 2024 Jan 8;24:36. doi: 10.1186/s12909-023-05012-7 (PMC10773098; doi:10.1186/s12909-023-05012-7)
Supplement: Supplementary file 1 — Additional file 1. Questionnaire for Research on offerings of mathematics series course. [file 12909_2023_5012_MOESM1_ESM.pdf]

Questionnaire for Research on offerings of mathematics series course

Respected teachers:

Hello! In the context of general health background, in order to better implement the role of the Mathematics series of courses in the process of cultivating medical and health talents, and to promote the healthy development of the medical mathematics curriculum system, the following research is made. The data of this research is only for the research of the provincial key teaching reform project "research and practice exploration of Mathematics curriculum system under the cultivation demand of new medical talents with quality as the priority and competence as the essential", and at the same time, it is also for laying the foundation for the promotion of the Ministry of Education's project of "practical research on Mathematics curriculum system of cultivation of pharmaceutical and healthcare talents under the background of big health" (which has already been sent to the Ministry of Education for review and approval). Your hard work is appreciated!

---Anhui Medical University project research group

April 26, 2021

Part I General Information

1. Your school name ____________________________

2. Your field of study ____________________ (for managerial positions: management; for pure teaching staff: teaching)

3. The name of the main course you teach ________________ (full-time researchers: research; full-time administrators: do not fill in)

4. Your age ________________

5. Your highest degree Doctor Degree☐ Master degree☐ Bachelor degree and below☐

6. Your job title Professor☐ Associate professor☐ Lecturer☐ Assistant☐

Part II Course Information

**Please read the names of the following majors carefully and place a √ after the appropriate option against the actual courses offered at your school.** Based on your worldview and perceptions, in conjunction with the needs of your major, place a ★ in the box (☐) after the name of the course that **should have been offered but in fact was not**. Place a × in the box (☐) after the name of the course **in the Mathematics series if you feel that it should not be offered in any major**. If there exists a major in your school not listed below that has been offered in the Mathematics series, please also add it to **the blank field for the name of the major** that follows.

| Name of major | Name of the course | Course setting | Curriculum attribute | Assessment method | Evaluation of effectiveness |
|---|---|---|---|---|---|
| Clinical Medicine （5+3） | Adanced mathematics□ | | Exam□ Investigate□ | Open-book□ Closed-book□ Online□ Offline□ | Good□ General□ Poor□ |
| | Linear algebra □ | | Exam□ Investigate□ | Open-book□ Closed-book□ Online□ Offline□ | Good□ General□ Poor□ |
| | Probability □ | | Exam□ Investigate□ | Open-book□ Closed-book□ Online□ Offline□ | Good□ General□ Poor□ |
| | Operational research□ | | Exam□ Investigate□ | Open-book□ Closed-book□ Online□ Offline□ | Good□ General□ Poor□ |
| | Function of a complex variable □ | | Exam□ Investigate□ | Open-book□ Closed-book□ Online□ Offline□ | Good□ General□ Poor□ |
| | Applied mathematics□ | | Exam□ Investigate□ | Open-book□ Closed-book□ Online□ Offline□ | Good□ General□ Poor□ |
| | Mathematics science class □ | | Exam□ Investigate□ | Open-book□ Closed-book□ Online□ Offline□ | Good□ General□ Poor□ |
| | | | | | |
| Clinical Medicine | Adanced mathematics□ | | Exam□ Investigate□ | Open-book□ Closed-book□ Online□ Offline□ | Good□ General□ Poor□ |
| | Linear algebra □ | | Exam□ Investigate□ | Open-book□ Closed-book□ Online□ Offline□ | Good□ General□ Poor□ |
| | Probability □ | | Exam□ Investigate□ | Open-book□ Closed-book□ Online□ Offline□ | Good□ General□ Poor□ |
| | Operational research□ | | Exam□ Investigate□ | Open-book□ Closed-book□ Online□ Offline□ | Good□ General□ Poor□ |
| | Function of a complex variable □ | | Exam□ Investigate□ | Open-book□ Closed-book□ Online□ Offline□ | Good□ General□ Poor□ |
| | Applied mathematics□ | | Exam□ Investigate□ | Open-book□ Closed-book□ Online□ Offline□ | Good□ General□ Poor□ |
| | Mathematics science class □ | | Exam□ Investigate□ | Open-book□ Closed-book□ Online□ Offline□ | Good□ General□ Poor□ |
| | | | | | |
| Aneasthesiology | Adanced mathematics□ | | Exam□ Investigate□ | Open-book□ Closed-book□ Online□ Offline□ | Good□ General□ Poor□ |
| | Linear algebra □ | | Exam□ Investigate□ | Open-book□ Closed-book□ Online□ Offline□ | Good□ General□ Poor□ |

| | | | | | |
|---|---|---|---|---|---|
| | Probability □ | | Exam□ Investigate□ | Open-book□ Closed-book□ Online□ Offline□ | Good□ General□ Poor□ |
| | Operational research□ | | Exam□ Investigate□ | Open-book□ Closed-book□ Online□ Offline□ | Good□ General□ Poor□ |
| | Function of a complex variable □ | | Exam□ Investigate□ | Open-book□ Closed-book□ Online□ Offline□ | Good□ General□ Poor□ |
| | Applied mathematics□ | | Exam□ Investigate□ | Open-book□ Closed-book□ Online□ Offline□ | Good□ General□ Poor□ |
| | Mathematics science class □ | | Exam□ Investigate□ | Open-book□ Closed-book□ Online□ Offline□ | Good□ General□ Poor□ |
| | | | | | |
| Medical Imaging | Adanced mathematics□ | | Exam□ Investigate□ | Open-book□ Closed-book□ Online□ Offline□ | Good□ General□ Poor□ |
| | Linear algebra □ | | Exam□ Investigate□ | Open-book□ Closed-book□ Online□ Offline□ | Good□ General□ Poor□ |
| | Probability □ | | Exam□ Investigate□ | Open-book□ Closed-book□ Online□ Offline□ | Good□ General□ Poor□ |
| | Operational research□ | | Exam□ Investigate□ | Open-book□ Closed-book□ Online□ Offline□ | Good□ General□ Poor□ |
| | Function of a complex variable □ | | Exam□ Investigate□ | Open-book□ Closed-book□ Online□ Offline□ | Good□ General□ Poor□ |
| | Applied mathematics□ | | Exam□ Investigate□ | Open-book□ Closed-book□ Online□ Offline□ | Good□ General□ Poor□ |
| | Mathematics science class □ | | Exam□ Investigate□ | Open-book□ Closed-book□ Online□ Offline□ | Good□ General□ Poor□ |
| | | | | | |
| Ophthalmology | Adanced mathematics□ | | Exam□ Investigate□ | Open-book□ Closed-book□ Online□ Offline□ | Good□ General□ Poor□ |
| | Linear algebra □ | | Exam□ Investigate□ | Open-book□ Closed-book□ Online□ Offline□ | Good□ General□ Poor□ |
| | Probability □ | | Exam□ Investigate□ | Open-book□ Closed-book□ Online□ Offline□ | Good□ General□ Poor□ |
| | Operational research□ | | Exam□ Investigate□ | Open-book□ Closed-book□ Online□ Offline□ | Good□ General□ Poor□ |
| | Function of a complex variable □ | | Exam□ Investigate□ | Open-book□ Closed-book□ Online□ Offline□ | Good□ General□ Poor□ |
| | Applied mathematics□ | | Exam□ Investigate□ | Open-book□ Closed-book□ Online□ Offline□ | Good□ General□ Poor□ |

| | | | | | |
|---|---|---|---|---|---|
| | Mathematics science class □ | | Exam□ Investigate□ | Open-book□ Closed-book□ Online□ Offline□ | Good□ General□ Poor□ |
| | | | | | |
| Psychiatry | Adanced mathematics□ | | Exam□ Investigate□ | Open-book□ Closed-book□ Online□ Offline□ | Good□ General□ Poor□ |
| | Linear algebra □ | | Exam□ Investigate□ | Open-book□ Closed-book□ Online□ Offline□ | Good□ General□ Poor□ |
| | Probability □ | | Exam□ Investigate□ | Open-book□ Closed-book□ Online□ Offline□ | Good□ General□ Poor□ |
| | Operational research□ | | Exam□ Investigate□ | Open-book□ Closed-book□ Online□ Offline□ | Good□ General□ Poor□ |
| | Function of a complex variable □ | | Exam□ Investigate□ | Open-book□ Closed-book□ Online□ Offline□ | Good□ General□ Poor□ |
| | Applied mathematics□ | | Exam□ Investigate□ | Open-book□ Closed-book□ Online□ Offline□ | Good□ General□ Poor□ |
| | Mathematics science class □ | | Exam□ Investigate□ | Open-book□ Closed-book□ Online□ Offline□ | Good□ General□ Poor□ |
| | | | | | |
| Radiological Medicine | Adanced mathematics□ | | Exam□ Investigate□ | Open-book□ Closed-book□ Online□ Offline□ | Good□ General□ Poor□ |
| | Linear algebra □ | | Exam□ Investigate□ | Open-book□ Closed-book□ Online□ Offline□ | Good□ General□ Poor□ |
| | Probability □ | | Exam□ Investigate□ | Open-book□ Closed-book□ Online□ Offline□ | Good□ General□ Poor□ |
| | Operational research□ | | Exam□ Investigate□ | Open-book□ Closed-book□ Online□ Offline□ | Good□ General□ Poor□ |
| | Function of a complex variable □ | | Exam□ Investigate□ | Open-book□ Closed-book□ Online□ Offline□ | Good□ General□ Poor□ |
| | Applied mathematics□ | | Exam□ Investigate□ | Open-book□ Closed-book□ Online□ Offline□ | Good□ General□ Poor□ |
| | Mathematics science class □ | | Exam□ Investigate□ | Open-book□ Closed-book□ Online□ Offline□ | Good□ General□ Poor□ |
| | | | | | |
| Postscript | | | | | |

| | | | | | |
|---|---|---|---|---|---|
| | | | | | |
| Paediatrics | Adanced mathematics□ | | Exam□ Investigate□ | Open-book□ Closed-book□ Online□ Offline□ | Good□ General□ Poor□ |
| | Linear algebra □ | | Exam□ Investigate□ | Open-book□ Closed-book□ Online□ Offline□ | Good□ General□ Poor□ |
| | Probability □ | | Exam□ Investigate□ | Open-book□ Closed-book□ Online□ Offline□ | Good□ General□ Poor□ |
| | Operational research□ | | Exam□ Investigate□ | Open-book□ Closed-book□ Online□ Offline□ | Good□ General□ Poor□ |
| | Function of a complex variable □ | | Exam□ Investigate□ | Open-book□ Closed-book□ Online□ Offline□ | Good□ General□ Poor□ |
| | Applied mathematics□ | | Exam□ Investigate□ | Open-book□ Closed-book□ Online□ Offline□ | Good□ General□ Poor□ |
| | Mathematics science class □ | | Exam□ Investigate□ | Open-book□ Closed-book□ Online□ Offline□ | Good□ General□ Poor□ |
| | | | | | |
| Basic Medicine | Adanced mathematics□ | | Exam□ Investigate□ | Open-book□ Closed-book□ Online□ Offline□ | Good□ General□ Poor□ |
| | Linear algebra □ | | Exam□ Investigate□ | Open-book□ Closed-book□ Online□ Offline□ | Good□ General□ Poor□ |
| | Probability □ | | Exam□ Investigate□ | Open-book□ Closed-book□ Online□ Offline□ | Good□ General□ Poor□ |
| | Operational research□ | | Exam□ Investigate□ | Open-book□ Closed-book□ Online□ Offline□ | Good□ General□ Poor□ |
| | Function of a complex variable □ | | Exam□ Investigate□ | Open-book□ Closed-book□ Online□ Offline□ | Good□ General□ Poor□ |
| | Applied mathematics□ | | Exam□ Investigate□ | Open-book□ Closed-book□ Online□ Offline□ | Good□ General□ Poor□ |
| | Mathematics science class □ | | Exam□ Investigate□ | Open-book□ Closed-book□ Online□ Offline□ | Good□ General□ Poor□ |
| | | | | | |
| Dental Medicine | Adanced mathematics□ | | Exam□ Investigate□ | Open-book□ Closed-book□ Online□ Offline□ | Good□ General□ Poor□ |
| | Linear algebra □ | | Exam□ Investigate□ | Open-book□ Closed-book□ Online□ Offline□ | Good□ General□ Poor□ |
| | Probability □ | | Exam□ Investigate□ | Open-book□ Closed-book□ Online□ Offline□ | Good□ General□ Poor□ |
| | Operational research□ | | Exam□ Investigate□ | Open-book□ Closed-book□ Online□ Offline□ | Good□ General□ Poor□ |

| | | | | | |
|---|---|---|---|---|---|
| | Function of a complex variable □ | | Exam□ Investigate□ | Open-book□ Closed-book□ Online□ Offline□ | Good□ General□ Poor□ |
| | Applied mathematics□ | | Exam□ Investigate□ | Open-book□ Closed-book□ Online□ Offline□ | Good□ General□ Poor□ |
| | Mathematics science class □ | | Exam□ Investigate□ | Open-book□ Closed-book□ Online□ Offline□ | Good□ General□ Poor□ |
| | | | | | |
| Medical Laboratory Technology | Adanced mathematics□ | | Exam□ Investigate□ | Open-book□ Closed-book□ Online□ Offline□ | Good□ General□ Poor□ |
| | Linear algebra □ | | Exam□ Investigate□ | Open-book□ Closed-book□ Online□ Offline□ | Good□ General□ Poor□ |
| | Probability □ | | Exam□ Investigate□ | Open-book□ Closed-book□ Online□ Offline□ | Good□ General□ Poor□ |
| | Operational research□ | | Exam□ Investigate□ | Open-book□ Closed-book□ Online□ Offline□ | Good□ General□ Poor□ |
| | Function of a complex variable □ | | Exam□ Investigate□ | Open-book□ Closed-book□ Online□ Offline□ | Good□ General□ Poor□ |
| | Applied mathematics□ | | Exam□ Investigate□ | Open-book□ Closed-book□ Online□ Offline□ | Good□ General□ Poor□ |
| | Mathematics science class □ | | Exam□ Investigate□ | Open-book□ Closed-book□ Online□ Offline□ | Good□ General□ Poor□ |
| | | | | | |
| Rehabilitation Therapy | Adanced mathematics□ | | Exam□ Investigate□ | Open-book□ Closed-book□ Online□ Offline□ | Good□ General□ Poor□ |
| | Linear algebra □ | | Exam□ Investigate□ | Open-book□ Closed-book□ Online□ Offline□ | Good□ General□ Poor□ |
| | Probability □ | | Exam□ Investigate□ | Open-book□ Closed-book□ Online□ Offline□ | Good□ General□ Poor□ |
| | Operational research□ | | Exam□ Investigate□ | Open-book□ Closed-book□ Online□ Offline□ | Good□ General□ Poor□ |
| | Function of a complex variable □ | | Exam□ Investigate□ | Open-book□ Closed-book□ Online□ Offline□ | Good□ General□ Poor□ |
| | Applied mathematics□ | | Exam□ Investigate□ | Open-book□ Closed-book□ Online□ Offline□ | Good□ General□ Poor□ |
| | Mathematics science class □ | | Exam□ Investigate□ | Open-book□ Closed-book□ Online□ Offline□ | Good□ General□ Poor□ |

| | | | | | |
|---|---|---|---|---|---|
| | | | | | |
| Postscript | | | | | |
| | | | | | |
| Health Inspection and Quarantine | Adanced mathematics□ | | Exam□ Investigate□ | Open-book□ Closed-book□ Online□ Offline□ | Good□ General□ Poor□ |
| | Linear algebra □ | | Exam□ Investigate□ | Open-book□ Closed-book□ Online□ Offline□ | Good□ General□ Poor□ |
| | Probability □ | | Exam□ Investigate□ | Open-book□ Closed-book□ Online□ Offline□ | Good□ General□ Poor□ |
| | Operational research□ | | Exam□ Investigate□ | Open-book□ Closed-book□ Online□ Offline□ | Good□ General□ Poor□ |
| | Function of a complex variable □ | | Exam□ Investigate□ | Open-book□ Closed-book□ Online□ Offline□ | Good□ General□ Poor□ |
| | Applied mathematics□ | | Exam□ Investigate□ | Open-book□ Closed-book□ Online□ Offline□ | Good□ General□ Poor□ |
| | Mathematics science class □ | | Exam□ Investigate□ | Open-book□ Closed-book□ Online□ Offline□ | Good□ General□ Poor□ |
| | | | | | |
| Medical Laboratory Technology (Chinese-foreign cooperative program) | Adanced mathematics□ | | Exam□ Investigate□ | Open-book□ Closed-book□ Online□ Offline□ | Good□ General□ Poor□ |
| | Linear algebra □ | | Exam□ Investigate□ | Open-book□ Closed-book□ Online□ Offline□ | Good□ General□ Poor□ |
| | Probability □ | | Exam□ Investigate□ | Open-book□ Closed-book□ Online□ Offline□ | Good□ General□ Poor□ |
| | Operational research□ | | Exam□ Investigate□ | Open-book□ Closed-book□ Online□ Offline□ | Good□ General□ Poor□ |
| | Function of a complex variable □ | | Exam□ Investigate□ | Open-book□ Closed-book□ Online□ Offline□ | Good□ General□ Poor□ |
| | Applied mathematics□ | | Exam□ Investigate□ | Open-book□ Closed-book□ Online□ Offline□ | Good□ General□ Poor□ |
| | Mathematics science class □ | | Exam□ Investigate□ | Open-book□ Closed-book□ Online□ Offline□ | Good□ General□ Poor□ |
| | | | | | |
| Food Hygiene and Nutrition | Adanced mathematics□ | | Exam□ Investigate□ | Open-book□ Closed-book□ Online□ Offline□ | Good□ General□ Poor□ |
| | Linear algebra □ | | Exam□ Investigate□ | Open-book□ Closed-book□ Online□ Offline□ | Good□ General□ Poor□ |

| | | | | | |
|---|---|---|---|---|---|
| | Probability □ | | Exam□ Investigate□ | Open-book□ Closed-book□ Online□ Offline□ | Good□ General□ Poor□ |
| | Operational research□ | | Exam□ Investigate□ | Open-book□ Closed-book□ Online□ Offline□ | Good□ General□ Poor□ |
| | Function of a complex variable □ | | Exam□ Investigate□ | Open-book□ Closed-book□ Online□ Offline□ | Good□ General□ Poor□ |
| | Applied mathematics□ | | Exam□ Investigate□ | Open-book□ Closed-book□ Online□ Offline□ | Good□ General□ Poor□ |
| | Mathematics science class □ | | Exam□ Investigate□ | Open-book□ Closed-book□ Online□ Offline□ | Good□ General□ Poor□ |
| | | | | | |
| Preventive Medicine | Adanced mathematics□ | | Exam□ Investigate□ | Open-book□ Closed-book□ Online□ Offline□ | Good□ General□ Poor□ |
| | Linear algebra □ | | Exam□ Investigate□ | Open-book□ Closed-book□ Online□ Offline□ | Good□ General□ Poor□ |
| | Probability □ | | Exam□ Investigate□ | Open-book□ Closed-book□ Online□ Offline□ | Good□ General□ Poor□ |
| | Operational research□ | | Exam□ Investigate□ | Open-book□ Closed-book□ Online□ Offline□ | Good□ General□ Poor□ |
| | Function of a complex variable □ | | Exam□ Investigate□ | Open-book□ Closed-book□ Online□ Offline□ | Good□ General□ Poor□ |
| | Applied mathematics□ | | Exam□ Investigate□ | Open-book□ Closed-book□ Online□ Offline□ | Good□ General□ Poor□ |
| | Mathematics science class □ | | Exam□ Investigate□ | Open-book□ Closed-book□ Online□ Offline□ | Good□ General□ Poor□ |
| | | | | | |
| Maternal and Child Health Sciences | Adanced mathematics□ | | Exam□ Investigate□ | Open-book□ Closed-book□ Online□ Offline□ | Good□ General□ Poor□ |
| | Linear algebra □ | | Exam□ Investigate□ | Open-book□ Closed-book□ Online□ Offline□ | Good□ General□ Poor□ |
| | Probability □ | | Exam□ Investigate□ | Open-book□ Closed-book□ Online□ Offline□ | Good□ General□ Poor□ |
| | Operational research□ | | Exam□ Investigate□ | Open-book□ Closed-book□ Online□ Offline□ | Good□ General□ Poor□ |
| | Function of a complex variable □ | | Exam□ Investigate□ | Open-book□ Closed-book□ Online□ Offline□ | Good□ General□ Poor□ |
| | Applied mathematics□ | | Exam□ Investigate□ | Open-book□ Closed-book□ Online□ Offline□ | Good□ General□ Poor□ |

| | | | | | |
|---|---|---|---|---|---|
| | Mathematics science class □ | | Exam□ Investigate□ | Open-book□ Closed-book□ Online□ Offline□ | Good□ General□ Poor□ |
| | | | | | |
| Postscript | | | | | |
| | | | | | |
| Pharmacy | Adanced mathematics□ | | Exam□ Investigate□ | Open-book□ Closed-book□ Online□ Offline□ | Good□ General□ Poor□ |
| | Linear algebra □ | | Exam□ Investigate□ | Open-book□ Closed-book□ Online□ Offline□ | Good□ General□ Poor□ |
| | Probability □ | | Exam□ Investigate□ | Open-book□ Closed-book□ Online□ Offline□ | Good□ General□ Poor□ |
| | Operational research□ | | Exam□ Investigate□ | Open-book□ Closed-book□ Online□ Offline□ | Good□ General□ Poor□ |
| | Function of a complex variable □ | | Exam□ Investigate□ | Open-book□ Closed-book□ Online□ Offline□ | Good□ General□ Poor□ |
| | Applied mathematics□ | | Exam□ Investigate□ | Open-book□ Closed-book□ Online□ Offline□ | Good□ General□ Poor□ |
| | Mathematics science class □ | | Exam□ Investigate□ | Open-book□ Closed-book□ Online□ Offline□ | Good□ General□ Poor□ |
| | | | | | |
| Clinical Pharmacy | Adanced mathematics□ | | Exam□ Investigate□ | Open-book□ Closed-book□ Online□ Offline□ | Good□ General□ Poor□ |
| | Linear algebra □ | | Exam□ Investigate□ | Open-book□ Closed-book□ Online□ Offline□ | Good□ General□ Poor□ |
| | Probability □ | | Exam□ Investigate□ | Open-book□ Closed-book□ Online□ Offline□ | Good□ General□ Poor□ |
| | Operational research□ | | Exam□ Investigate□ | Open-book□ Closed-book□ Online□ Offline□ | Good□ General□ Poor□ |
| | Function of a complex variable □ | | Exam□ Investigate□ | Open-book□ Closed-book□ Online□ Offline□ | Good□ General□ Poor□ |
| | Applied mathematics□ | | Exam□ Investigate□ | Open-book□ Closed-book□ Online□ Offline□ | Good□ General□ Poor□ |
| | Mathematics science class □ | | Exam□ Investigate□ | Open-book□ Closed-book□ Online□ Offline□ | Good□ General□ Poor□ |
| | | | | | |

| | | | | | |
|---|---|---|---|---|---|
| Chinese Herbal Medicine | Adanced mathematics□ | | Exam□ Investigate□ | Open-book□ Closed-book□ Online□ Offline□ | Good□ General□ Poor□ |
| | Linear algebra □ | | Exam□ Investigate□ | Open-book□ Closed-book□ Online□ Offline□ | Good□ General□ Poor□ |
| | Probability □ | | Exam□ Investigate□ | Open-book□ Closed-book□ Online□ Offline□ | Good□ General□ Poor□ |
| | Operational research□ | | Exam□ Investigate□ | Open-book□ Closed-book□ Online□ Offline□ | Good□ General□ Poor□ |
| | Function of a complex variable □ | | Exam□ Investigate□ | Open-book□ Closed-book□ Online□ Offline□ | Good□ General□ Poor□ |
| | Applied mathematics□ | | Exam□ Investigate□ | Open-book□ Closed-book□ Online□ Offline□ | Good□ General□ Poor□ |
| | Mathematics science class □ | | Exam□ Investigate□ | Open-book□ Closed-book□ Online□ Offline□ | Good□ General□ Poor□ |
| | | | | | |
| Nursing | Adanced mathematics□ | | Exam□ Investigate□ | Open-book□ Closed-book□ Online□ Offline□ | Good□ General□ Poor□ |
| | Linear algebra □ | | Exam□ Investigate□ | Open-book□ Closed-book□ Online□ Offline□ | Good□ General□ Poor□ |
| | Probability □ | | Exam□ Investigate□ | Open-book□ Closed-book□ Online□ Offline□ | Good□ General□ Poor□ |
| | Operational research□ | | Exam□ Investigate□ | Open-book□ Closed-book□ Online□ Offline□ | Good□ General□ Poor□ |
| | Function of a complex variable □ | | Exam□ Investigate□ | Open-book□ Closed-book□ Online□ Offline□ | Good□ General□ Poor□ |
| | Applied mathematics□ | | Exam□ Investigate□ | Open-book□ Closed-book□ Online□ Offline□ | Good□ General□ Poor□ |
| | Mathematics science class □ | | Exam□ Investigate□ | Open-book□ Closed-book□ Online□ Offline□ | Good□ General□ Poor□ |
| | | | | | |
| Midwifery | Adanced mathematics□ | | Exam□ Investigate□ | Open-book□ Closed-book□ Online□ Offline□ | Good□ General□ Poor□ |
| | Linear algebra □ | | Exam□ Investigate□ | Open-book□ Closed-book□ Online□ Offline□ | Good□ General□ Poor□ |
| | Probability □ | | Exam□ Investigate□ | Open-book□ Closed-book□ Online□ Offline□ | Good□ General□ Poor□ |
| | Operational research□ | | Exam□ Investigate□ | Open-book□ Closed-book□ Online□ Offline□ | Good□ General□ Poor□ |

| | | | | | |
|---|---|---|---|---|---|
| | Function of a complex variable □ | | Exam□ Investigate□ | Open-book□ Closed-book□ Online□ Offline□ | Good□ General□ Poor□ |
| | Applied mathematics□ | | Exam□ Investigate□ | Open-book□ Closed-book□ Online□ Offline□ | Good□ General□ Poor□ |
| | Mathematics science class □ | | Exam□ Investigate□ | Open-book□ Closed-book□ Online□ Offline□ | Good□ General□ Poor□ |
| | | | | | |
| Postscript | | | | | |
| | | | | | |
| Biological Sciences | Adanced mathematics□ | | Exam□ Investigate□ | Open-book□ Closed-book□ Online□ Offline□ | Good□ General□ Poor□ |
| | Linear algebra □ | | Exam□ Investigate□ | Open-book□ Closed-book□ Online□ Offline□ | Good□ General□ Poor□ |
| | Probability □ | | Exam□ Investigate□ | Open-book□ Closed-book□ Online□ Offline□ | Good□ General□ Poor□ |
| | Operational research□ | | Exam□ Investigate□ | Open-book□ Closed-book□ Online□ Offline□ | Good□ General□ Poor□ |
| | Function of a complex variable □ | | Exam□ Investigate□ | Open-book□ Closed-book□ Online□ Offline□ | Good□ General□ Poor□ |
| | Applied mathematics□ | | Exam□ Investigate□ | Open-book□ Closed-book□ Online□ Offline□ | Good□ General□ Poor□ |
| | Mathematics science class □ | | Exam□ Investigate□ | Open-book□ Closed-book□ Online□ Offline□ | Good□ General□ Poor□ |
| | | | | | |
| Biotechnology | Adanced mathematics□ | | Exam□ Investigate□ | Open-book□ Closed-book□ Online□ Offline□ | Good□ General□ Poor□ |
| | Linear algebra □ | | Exam□ Investigate□ | Open-book□ Closed-book□ Online□ Offline□ | Good□ General□ Poor□ |
| | Probability □ | | Exam□ Investigate□ | Open-book□ Closed-book□ Online□ Offline□ | Good□ General□ Poor□ |
| | Operational research□ | | Exam□ Investigate□ | Open-book□ Closed-book□ Online□ Offline□ | Good□ General□ Poor□ |
| | Function of a complex variable □ | | Exam□ Investigate□ | Open-book□ Closed-book□ Online□ Offline□ | Good□ General□ Poor□ |

| | | | | | |
|---|---|---|---|---|---|
| | Applied mathematics□ | | Exam□ Investigate□ | Open-book□ Closed-book□ Online□ Offline□ | Good□ General□ Poor□ |
| | Mathematics science class □ | | Exam□ Investigate□ | Open-book□ Closed-book□ Online□ Offline□ | Good□ General□ Poor□ |
| | | | | | |
| Applied Psychology | Adanced mathematics□ | | Exam□ Investigate□ | Open-book□ Closed-book□ Online□ Offline□ | Good□ General□ Poor□ |
| | Linear algebra □ | | Exam□ Investigate□ | Open-book□ Closed-book□ Online□ Offline□ | Good□ General□ Poor□ |
| | Probability □ | | Exam□ Investigate□ | Open-book□ Closed-book□ Online□ Offline□ | Good□ General□ Poor□ |
| | Operational research□ | | Exam□ Investigate□ | Open-book□ Closed-book□ Online□ Offline□ | Good□ General□ Poor□ |
| | Function of a complex variable □ | | Exam□ Investigate□ | Open-book□ Closed-book□ Online□ Offline□ | Good□ General□ Poor□ |
| | Applied mathematics□ | | Exam□ Investigate□ | Open-book□ Closed-book□ Online□ Offline□ | Good□ General□ Poor□ |
| | Mathematics science class □ | | Exam□ Investigate□ | Open-book□ Closed-book□ Online□ Offline□ | Good□ General□ Poor□ |
| | | | | | |
| Public Administration | Adanced mathematics□ | | Exam□ Investigate□ | Open-book□ Closed-book□ Online□ Offline□ | Good□ General□ Poor□ |
| | Linear algebra □ | | Exam□ Investigate□ | Open-book□ Closed-book□ Online□ Offline□ | Good□ General□ Poor□ |
| | Probability □ | | Exam□ Investigate□ | Open-book□ Closed-book□ Online□ Offline□ | Good□ General□ Poor□ |
| | Operational research□ | | Exam□ Investigate□ | Open-book□ Closed-book□ Online□ Offline□ | Good□ General□ Poor□ |
| | Function of a complex variable □ | | Exam□ Investigate□ | Open-book□ Closed-book□ Online□ Offline□ | Good□ General□ Poor□ |
| | Applied mathematics□ | | Exam□ Investigate□ | Open-book□ Closed-book□ Online□ Offline□ | Good□ General□ Poor□ |
| | Mathematics science class □ | | Exam□ Investigate□ | Open-book□ Closed-book□ Online□ Offline□ | Good□ General□ Poor□ |
| | | | | | |
| Labour and Social | Adanced mathematics□ | | Exam□ Investigate□ | Open-book□ Closed-book□ Online□ Offline□ | Good□ General□ Poor□ |

| | | | | | |
|---|---|---|---|---|---|
| Security | Linear algebra □ | | Exam□ Investigate□ | Open-book□ Closed-book□ Online□ Offline□ | Good□ General□ Poor□ |
| | Probability □ | | Exam□ Investigate□ | Open-book□ Closed-book□ Online□ Offline□ | Good□ General□ Poor□ |
| | Operational research□ | | Exam□ Investigate□ | Open-book□ Closed-book□ Online□ Offline□ | Good□ General□ Poor□ |
| | Function of a complex variable □ | | Exam□ Investigate□ | Open-book□ Closed-book□ Online□ Offline□ | Good□ General□ Poor□ |
| | Applied mathematics□ | | Exam□ Investigate□ | Open-book□ Closed-book□ Online□ Offline□ | Good□ General□ Poor□ |
| | Mathematics science class □ | | Exam□ Investigate□ | Open-book□ Closed-book□ Online□ Offline□ | Good□ General□ Poor□ |
| | | | | | |
| Postscript | | | | | |
| | | | | | |
| Health Services Management | Adanced mathematics□ | | Exam□ Investigate□ | Open-book□ Closed-book□ Online□ Offline□ | Good□ General□ Poor□ |
| | Linear algebra □ | | Exam□ Investigate□ | Open-book□ Closed-book□ Online□ Offline□ | Good□ General□ Poor□ |
| | Probability □ | | Exam□ Investigate□ | Open-book□ Closed-book□ Online□ Offline□ | Good□ General□ Poor□ |
| | Operational research□ | | Exam□ Investigate□ | Open-book□ Closed-book□ Online□ Offline□ | Good□ General□ Poor□ |
| | Function of a complex variable □ | | Exam□ Investigate□ | Open-book□ Closed-book□ Online□ Offline□ | Good□ General□ Poor□ |
| | Applied mathematics□ | | Exam□ Investigate□ | Open-book□ Closed-book□ Online□ Offline□ | Good□ General□ Poor□ |
| | Mathematics science class □ | | Exam□ Investigate□ | Open-book□ Closed-book□ Online□ Offline□ | Good□ General□ Poor□ |
| | | | | | |
| Information Management and | Adanced mathematics□ | | Exam□ Investigate□ | Open-book□ Closed-book□ Online□ Offline□ | Good□ General□ Poor□ |
| | Linear algebra □ | | Exam□ Investigate□ | Open-book□ Closed-book□ Online□ Offline□ | Good□ General□ Poor□ |
| | Probability □ | | Exam□ Investigate□ | Open-book□ Closed-book□ Online□ Offline□ | Good□ General□ Poor□ |

| | | | | | |
|---|---|---|---|---|---|
| Information System | Operational research□ | | Exam□ Investigate□ | Open-book□ Closed-book□ Online□ Offline□ | Good□ General□ Poor□ |
| | Function of a complex variable □ | | Exam□ Investigate□ | Open-book□ Closed-book□ Online□ Offline□ | Good□ General□ Poor□ |
| | Applied mathematics□ | | Exam□ Investigate□ | Open-book□ Closed-book□ Online□ Offline□ | Good□ General□ Poor□ |
| | Mathematics science class □ | | Exam□ Investigate□ | Open-book□ Closed-book□ Online□ Offline□ | Good□ General□ Poor□ |
| | | | | | |
| Medical Information Engineering | Adanced mathematics□ | | Exam□ Investigate□ | Open-book□ Closed-book□ Online□ Offline□ | Good□ General□ Poor□ |
| | Linear algebra □ | | Exam□ Investigate□ | Open-book□ Closed-book□ Online□ Offline□ | Good□ General□ Poor□ |
| | Probability □ | | Exam□ Investigate□ | Open-book□ Closed-book□ Online□ Offline□ | Good□ General□ Poor□ |
| | Operational research□ | | Exam□ Investigate□ | Open-book□ Closed-book□ Online□ Offline□ | Good□ General□ Poor□ |
| | Function of a complex variable □ | | Exam□ Investigate□ | Open-book□ Closed-book□ Online□ Offline□ | Good□ General□ Poor□ |
| | Applied mathematics□ | | Exam□ Investigate□ | Open-book□ Closed-book□ Online□ Offline□ | Good□ General□ Poor□ |
| | Mathematics science class □ | | Exam□ Investigate□ | Open-book□ Closed-book□ Online□ Offline□ | Good□ General□ Poor□ |
| | | | | | |
| Biomedical Engineering | Adanced mathematics□ | | Exam□ Investigate□ | Open-book□ Closed-book□ Online□ Offline□ | Good□ General□ Poor□ |
| | Linear algebra □ | | Exam□ Investigate□ | Open-book□ Closed-book□ Online□ Offline□ | Good□ General□ Poor□ |
| | Probability □ | | Exam□ Investigate□ | Open-book□ Closed-book□ Online□ Offline□ | Good□ General□ Poor□ |
| | Operational research□ | | Exam□ Investigate□ | Open-book□ Closed-book□ Online□ Offline□ | Good□ General□ Poor□ |
| | Function of a complex variable □ | | Exam□ Investigate□ | Open-book□ Closed-book□ Online□ Offline□ | Good□ General□ Poor□ |
| | Applied mathematics□ | | Exam□ Investigate□ | Open-book□ Closed-book□ Online□ Offline□ | Good□ General□ Poor□ |
| | Mathematics science | | Exam□ Investigate□ | Open-book□ Closed-book□ Online□ Offline□ | Good□ General□ Poor□ |

| | | | | | |
|---|---|---|---|---|---|
| | class ☐ | | | | |
| | | | | | |
| Law | Adanced mathematics☐ | | Exam☐ Investigate☐ | Open-book☐ Closed-book☐ Online☐ Offline☐ | Good☐ General☐ Poor☐ |
| | Linear algebra ☐ | | Exam☐ Investigate☐ | Open-book☐ Closed-book☐ Online☐ Offline☐ | Good☐ General☐ Poor☐ |
| | Probability ☐ | | Exam☐ Investigate☐ | Open-book☐ Closed-book☐ Online☐ Offline☐ | Good☐ General☐ Poor☐ |
| | Operational research☐ | | Exam☐ Investigate☐ | Open-book☐ Closed-book☐ Online☐ Offline☐ | Good☐ General☐ Poor☐ |
| | Function of a complex variable ☐ | | Exam☐ Investigate☐ | Open-book☐ Closed-book☐ Online☐ Offline☐ | Good☐ General☐ Poor☐ |
| | Applied mathematics☐ | | Exam☐ Investigate☐ | Open-book☐ Closed-book☐ Online☐ Offline☐ | Good☐ General☐ Poor☐ |
| | Mathematics science class ☐ | | Exam☐ Investigate☐ | Open-book☐ Closed-book☐ Online☐ Offline☐ | Good☐ General☐ Poor☐ |
| | | | | | |
| Postscript | | | | | |
| Note: No majors are listed (optional) | | | | | |
| | Adanced mathematics☐ | | Exam☐ Investigate☐ | Open-book☐ Closed-book☐ Online☐ Offline☐ | Good☐ General☐ Poor☐ |
| | Linear algebra ☐ | | Exam☐ Investigate☐ | Open-book☐ Closed-book☐ Online☐ Offline☐ | Good☐ General☐ Poor☐ |
| | Probability ☐ | | Exam☐ Investigate☐ | Open-book☐ Closed-book☐ Online☐ Offline☐ | Good☐ General☐ Poor☐ |
| | Operational research☐ | | Exam☐ Investigate☐ | Open-book☐ Closed-book☐ Online☐ Offline☐ | Good☐ General☐ Poor☐ |
| | Function of a complex variable ☐ | | Exam☐ Investigate☐ | Open-book☐ Closed-book☐ Online☐ Offline☐ | Good☐ General☐ Poor☐ |
| | Applied mathematics☐ | | Exam☐ Investigate☐ | Open-book☐ Closed-book☐ Online☐ Offline☐ | Good☐ General☐ Poor☐ |
| | Mathematics science class ☐ | | Exam☐ Investigate☐ | Open-book☐ Closed-book☐ Online☐ Offline☐ | Good☐ General☐ Poor☐ |
| | | | | | |

| | | | | | |
|---|---|---|---|---|---|
| | Adanced mathematics□ | | Exam□ Investigate□ | Open-book□ Closed-book□ Online□ Offline□ | Good□ General□ Poor□ |
| | Linear algebra □ | | Exam□ Investigate□ | Open-book□ Closed-book□ Online□ Offline□ | Good□ General□ Poor□ |
| | Probability □ | | Exam□ Investigate□ | Open-book□ Closed-book□ Online□ Offline□ | Good□ General□ Poor□ |
| | Operational research□ | | Exam□ Investigate□ | Open-book□ Closed-book□ Online□ Offline□ | Good□ General□ Poor□ |
| | Function of a complex variable □ | | Exam□ Investigate□ | Open-book□ Closed-book□ Online□ Offline□ | Good□ General□ Poor□ |
| | Applied mathematics□ | | Exam□ Investigate□ | Open-book□ Closed-book□ Online□ Offline□ | Good□ General□ Poor□ |
| | Mathematics science class □ | | Exam□ Investigate□ | Open-book□ Closed-book□ Online□ Offline□ | Good□ General□ Poor□ |

## Part III Cognitive information

Based on your opinion, complete the following single-choice questions. Type √ on the options you agree with, indicate the degree of agreement or disagreement., which completely disagree with the selection of 1, basically disagree with the selection of 2, neither agree nor disagree with the selection of 3, basically agree with the selection of 4, completely agree with the selection of 5.

| Serial number | Question | Completely disagree ⟷ Completely agree | | | | |
|---|---|---|---|---|---|---|
| **1** | The contents of current medical mathematics textbook can meet the needs of professional requirements | 1 | 2 | 3 | 4 | 5 |
| **2** | The reform of medical mathematics teaching fully meets the cultivation of medical talents and can realize the integration of teaching and research | 1 | 2 | 3 | 4 | 5 |
| **3** | The contemporary process of teaching medical mathematics covers a certain number of on-site laboratory or social practice classes | 1 | 2 | 3 | 4 | 5 |
| **4** | The current medical mathematics teaching content is out of touch with the actual needs of professionals | 1 | 2 | 3 | 4 | 5 |
| **5** | The current curriculum system of medical colleges is inadequate and needs to be further integrated | 1 | 2 | 3 | 4 | 5 |
